# Fulminant demyelinating disease of the central nervous system effectively treated with a combination of decompressive craniectomy and immunotherapy: A case report and literature review

**DOI:** 10.1002/ccr3.9059

**Published:** 2024-07-09

**Authors:** Toshihiro Ide, Ryo Ebashi, Makoto Eriguchi, Shinichi Aishima, Tatsuya Abe, Hideo Hara

**Affiliations:** ^1^ Division of Neurology, Department of Internal Medicine, Faculty of Medicine Saga University Saga Japan; ^2^ Department of Neurosurgery, Faculty of Medicine Saga University Saga Japan; ^3^ Department of Pathology and Microbiology, Faculty of Medicine Saga University Saga Japan

**Keywords:** brain herniation, decompressive craniectomy, fulminant demyelinating disease of the central nervous system, IL‐6, immunotherapy

## Abstract

**Key Clinical Message:**

Accurately identifying fulminant demyelinating diseases is important for sudden onset of asymmetric cerebral white matter lesions with mass effect. Initially, immunotherapy should be administered; however, surgical intervention should be performed with poor response to medical management and evident signs of cerebral herniation.

**Abstract:**

A case of fulminant demyelinating disease of the central nervous system that required decompressive craniectomy 8 days after symptom onset is presented. The patient recovered without sequelae after a combination of neurosurgery and immunotherapy with steroids and has remained relapse‐free for 4 years.

## 1 INTRODUCTION

Fulminant demyelinating disease is a rare group of inflammatory demyelinating disorders that rapidly progress to disability within days to weeks, eventually requiring hospitalization and aggressive treatment for acute attacks.^1^ The present patient developed brain herniation within 8 days after symptom onset, but recovered without neurological sequelae after decompressive craniectomy and immunotherapy with steroids. Nevertheless, there have been few case reports of decompressive craniectomy for fulminant demyelinating disease, and the present case report aimed to clarify the clinical characteristics of such cases along with a review of the literature.

## 2 CASE HISTORY/EXAMINATION

**FIGURE 1:**
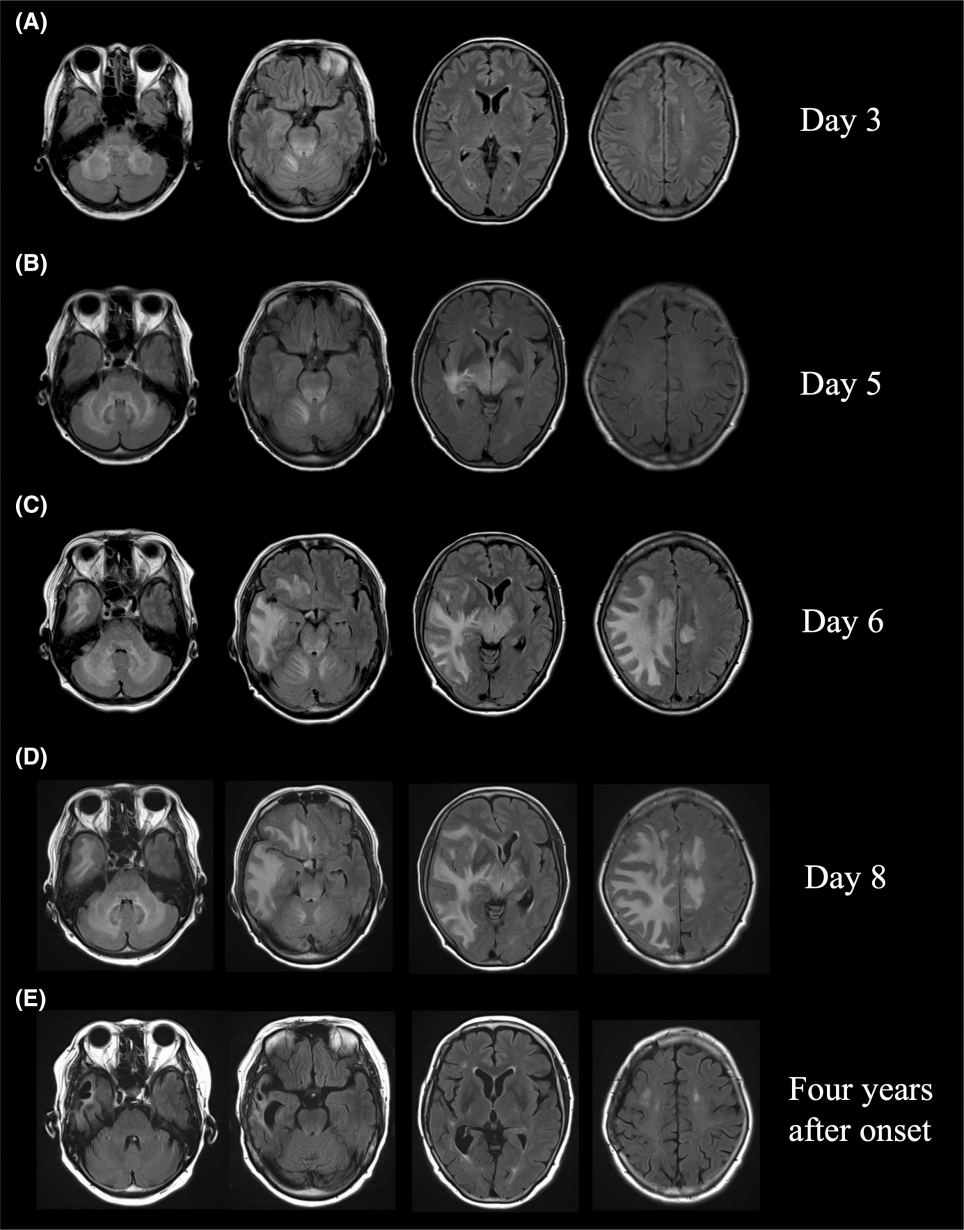
Brain MRI. Fluid attenuated inversion recovery (FLAIR) images show T2 high‐signal intensity lesions around the dentate nuclei, cerebellar peduncles, the right ventral side of the medulla oblongata, and tegmentum (Day 3, A). A new lesion is detected in the right temporal lobe (Day 5, B). The lesions expand rapidly, and extensive T2 high‐signal intensity lesions are observed in the bilateral cerebellar hemispheres, brainstem, bilateral thalami, right basal ganglia, right posterior limb of the internal capsule, and subcortical cerebral hemispheres. The abnormal signal area also extends to the left cingulate gyrus via the body of the corpus callosum. The lesion has a mass effect; the right ventricle is slightly deformed, and the median structure is deviated to the left (Day 6, C). The lesion has enlarged further, and the brain swelling is aggravated (Day 8, D). The brain swelling has improved, and the lesion has shrunk (4 years after onset, E).

A 63‐year‐old woman with no history of prior viral infections or vaccinations developed a headache in mid‐October 2018. On the following day, she became lightheaded and developed nausea, and she visited a local general hospital. On medical examination, her consciousness was clear, and she had no paralysis. However, she had diplopia and ataxia of both upper limbs. Magnetic resonance imaging (MRI) of the brain showed T2 high‐signal intensity lesions around the dentate nuclei of bilateral cerebellar hemispheres to the cerebellar peduncles, the right ventral side of the medulla oblongata, and the tegmentum (Figure 1A). On the 5th day, a mild disturbance of consciousness and paralysis of the left upper limb appeared, and MRI of the brain showed a new lesion in the right temporal lobe (Figure 1B). On the 6th day, she was urgently transferred to our hospital for further examination and treatment.

On admission, her temperature was 36.8°C, pulse was 63 beats/min, blood pressure was 146/88 mmHg, respiratory rate was 17 breaths/min, and general physical examination showed no abnormalities of note. On neurological examination, mild impairment of consciousness (Glasgow Coma Scale (GCS) E4V4M6), left hemiparesis (MMT 3), and ataxia of the extremities were evident. Meningeal stiffness was also observed. Blood tests showed mild inflammatory findings, with white blood cell count of 11,100 cells/μL and C‐reactive protein (CRP) of 1.26 mg/dL, but liver function, renal function, electrolytes, and coagulation function were all normal. Serum rheumatoid factor, anti‐nuclear antibody, anti‐SS‐A antibody, anti‐SS‐B antibody, myeloperoxidase‐anti‐neutrophil cytoplasmic antibody (MPO‐ANCA), serine proteinase3‐anti‐neutrophil cytoplasmic antibody (PR3‐ANCA), and other autoimmune markers were all negative. Anti‐aquaporin4‐antibody (anti‐AQP4 antibody) was negative, measured by both an enzyme‐linked immunosorbent assay (ELISA) and a cell‐based assay (CBA). Anti‐MOG antibody was also negative by a CBA. A cerebrospinal fluid (CSF) examination showed a normal initial pressure of 11 cmH_2_0 and a mildly elevated cell count with mononuclear cell predominance (50 cells/μL). CSF protein, β2‐microglobulin, and IgG were elevated at 96 mg/dL, 1.71 μg/mL, and 10.7 mg/dL, respectively. Myelin basic protein (MBP) was markedly elevated at 33,000 pg/mL. The IgG index was 0.64. There were no atypical cells on CSF cytology. The interleukin 6 (IL‐6) level in the CSF was markedly elevated at 1410 pg/mL. The electrocardiogram and chest X‐ray showed no abnormalities. MRI of the brain showed extensive T2 high‐signal intensity lesions in the bilateral cerebellar hemispheres, brainstem, bilateral thalami, right basal ganglia, right posterior limb of the internal capsule, and subcortical cerebral hemispheres. The abnormal signal area also extended to the left cingulate gyrus via the body of the corpus callosum. The lesion had a mass effect, with the right ventricle slightly deformed and the median structures deviated to the left (Figure 1C). A faint contrast effect was seen at the margins of the bilateral frontal lobe lesions. No lesion was found on spinal cord MRI.

## 3 DIFFERENTIAL DIAGNOSIS, INVESTIGATIONS AND TREATMENT

**FIGURE 2:**
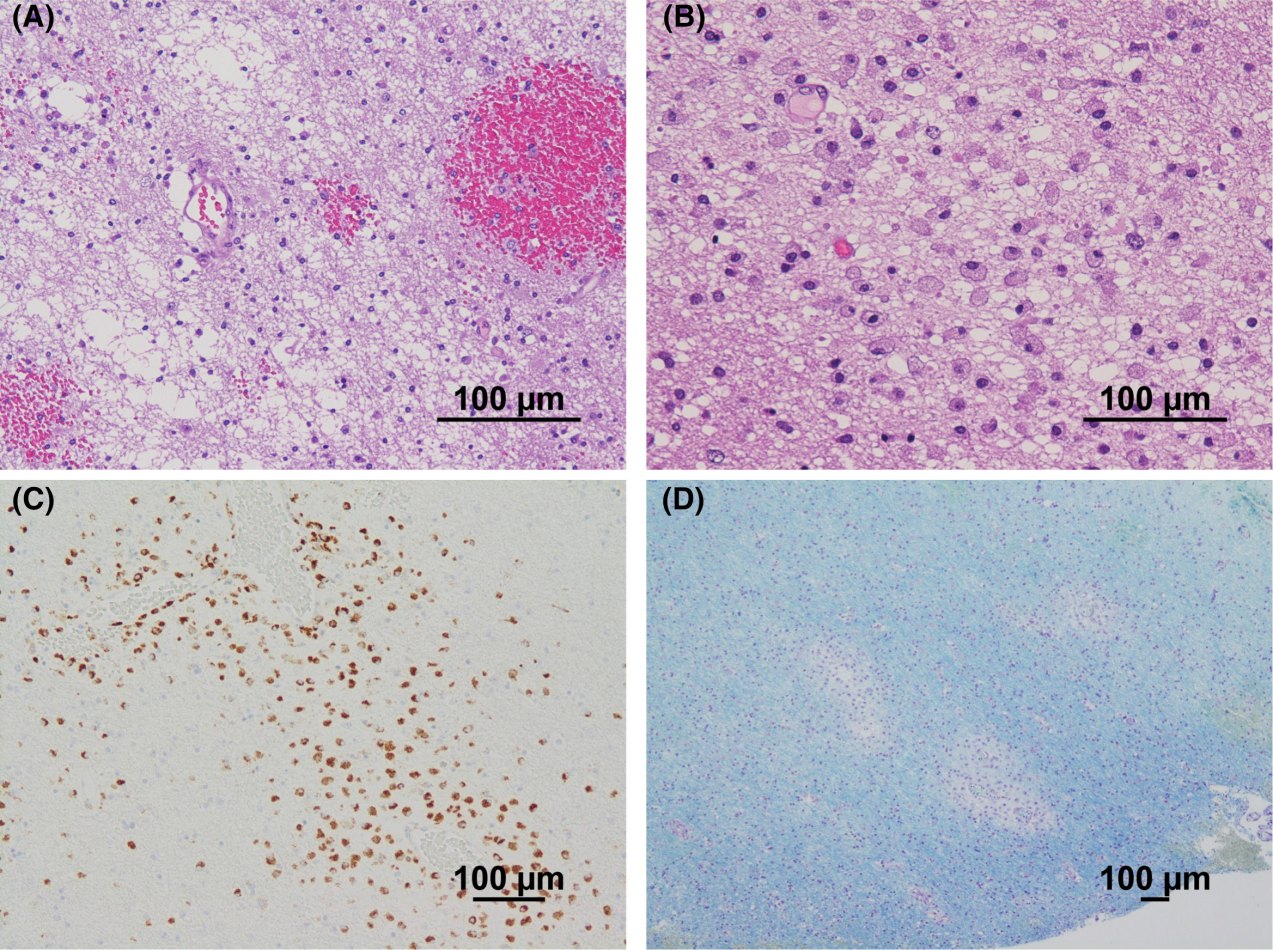
Brain biopsy specimens obtained from the right temporal lobe. Small haemorrhagic foci are scattered in the cerebral white matter (A). The white matter has been infiltrated with foamy macrophages (B). CD68 immunohistochemical staining is positive for histiocytes in the cerebral white matter (C). Demyelinating plaques with decreased density and staining of myelinated sheaths are seen on Klüver–Barrera staining (D). These findings suggest demyelinating disease. (A, B, hematoxylin and eosin staining, scale bar = 100 μm; C, CD68 immunostaining, scale bar = 100 μm; D, Klüver–Barrera staining, scale bar = 100 μm).

The rapid appearance of the white matter‐dominant lesions required a distinction between an inflammatory demyelinating disease of the central nervous system (CNS) and drug‐induced, toxic, or metabolic encephalopathy. However, the medical history and laboratory findings of the patient did not support the latter etiology. Furthermore, if the bilateral cerebellar hemispheres, brainstem, and bilateral thalami were involved, the asymmetric nature of cerebral white matter lesions with a pronounced mass effect and the marked increase in MBP in the CSF strongly suggested fulminant inflammatory demyelinating disease of the CNS. Therefore, steroid pulse therapy (methylprednisolone 1 g/day for 5 days) was started on the 6th day, but the disturbance of consciousness gradually worsened despite the initiation of steroids, and a follow‐up MRI showed further worsening of the brain swelling (Figure 1D). On the 8th day, her level of consciousness decreased to GCS E1V2M4, and she appeared to have developed brain herniation; emergency decompressive craniectomy was performed on the same day. At the time of craniotomy, a brain biopsy from the right temporal lobe was performed for diagnosis. The brain biopsy specimens showed small haemorrhagic foci in the cerebral white matter (Figure 2A) and infiltration of CD68‐positive foamy macrophages around the perivascular area (Figure 2B,C). Klüver–Barrera staining showed demyelinating plaques around the perivascular area (Figure 2D). These pathological findings were consistent with acute demyelinating encephalomyelitis (ADEM). After the decompressive craniectomy, immunotherapy with steroids was continued.

## 4 OUTCOME AND FOLLOW‐UP

The patient required mechanical ventilation, but she was extubated on the 20th day, and she was able to follow instructions. After extubation, she was able to speak without aphasia. Following the steroid pulse therapy, oral prednisolone 50 mg/day was started, which was maintained for 2 weeks and then gradually decreased by 5 mg/week. The left hemiplegia recovered gradually, and gait training was started on the 36th day of the disease; she was able to walk on her own within 2 months from disease onset. Cranioplasty was performed on the 42nd day after improvement of brain swelling and herniation on brain MRI. After tapering, prednisolone was maintained at 10 mg/day. The left hemiplegia recovered completely, and she was discharged home on the 122nd day. For 4 years after discharge, she has been clinically and radiologically free of recurrence (Figure 1E).

## 5 DISCUSSION

Fulminant inflammatory demyelinating disease of the CNS is a rare group of diseases in which immune‐mediated destruction of myelin occurs, but the clinical course, histopathology, and imaging findings differ from those of classical multiple sclerosis (MS). They include ADEM and its variants, acute haemorrhagic leukoencephalitis (AHL or Hurst disease), severe relapses of MS, variants of MS (tumefactive MS, Marburg variant, Baló's concentric sclerosis, myelinoclastic diffuse sclerosis), and neuromyelitis optica (NMO)‐spectrum disorders.^1^ In the present case, since the patient met the diagnostic criteria for ADEM and had no recurrence for 4 years, she was diagnosed with monophasic ADEM as a clinical subtype of ADEM.^2^ ADEM is an immune‐mediated demyelinating disease of the CNS that commonly affects children and young adults.^3^ The radiological features of ADEM include multifocal and diffuse white matter lesions with bilateral thalamic and bilateral basal ganglia involvement, which often affects the cerebellum and the brainstem.^1^ The imaging findings in our case were also compatible with ADEM. The hyperacute form of ADEM accounts for 2% of cases and is associated with rapid progression of symptoms, malignant brain edema, and a high mortality rate.^4^

The interesting feature of this case is that, although the patient presented with rapid cerebral herniation due to fulminant demyelination, she recovered with no neurological sequelae after treatment by a combination of decompressive craniectomy and immunotherapy. There are a few reports of cases requiring decompressive craniectomy for autoimmune diseases of the CNS, including demyelinating diseases, primary angiitis of the CNS,^5^ and neuropsychiatric systemic lupus erythematosus.^6^ The reports of fulminant demyelinating disease of the CNS requiring decompressive craniectomy are summarized in Table 1. So far, 17 cases have been reported (including the present case), with 8 cases of ADEM,^7^, ^8^, ^9^, ^10^, ^11^, ^12^, ^13^ 4 cases of AHL,^14^, ^15^, ^16^, ^17^ 3 cases of TDLs,^18^, ^19^, ^20^ and 2 cases of the Marburg variant of MS.^21^, ^22^ The age of onset ranged widely from 1 to 63 years, but 16 of 17 cases were in their 50s or younger, and 4 were children. The age of onset was the oldest in the present case. In addition, the incidence was higher in females (12 of 17 cases). Prior infections were known in 9 cases, and most of them were respiratory tract infections such as upper respiratory tract infection and pneumonia (including 2 cases of mycoplasma infection). In addition, ADEM and AHL were often accompanied by prior infection (9 of 12 cases). Steroids were the most commonly used immunotherapy in combination with decompressive craniectomy (all 16 cases with information available). Intravenous methylprednisolone (IVMP) was the most common, with 8 cases. Intravenous immunoglobulin (IVIG) and plasma apheresis therapy were also used in some cases. The outcome was complete recovery in 5 cases, neurological sequelae in 10 cases, and death in 1 case. Of the 16 cases—including the present case and the cases with information available—most (14/16) initially underwent immunotherapy. For the remaining cases, only one underwent immunotherapy after decompressive craniectomy, whereas another underwent both treatments simultaneously. In cases where immunotherapy was administered first, surgical intervention was subsequently performed if the response to medical management was inadequate, specifically in cases with (1) deteriorating consciousness, (2) increased intracranial pressure, and (3) worsening radiological findings. Previous reviews on fulminant demyelinating diseases emphasized that immunotherapy should be the first line of treatment, with decompressive craniectomy considered thereafter if the response to medical management was poor.^1^ Therefore, immunotherapy with steroids should be initially administered in fulminant demyelinating diseases aiming to reduce inflammation and edema, although decompressive craniectomy should be performed in cases that present signs of cerebral herniation.

**TABLE 1:** Clinical characteristics of reported cases of decompressive craniectomy for fulminant demyelinating disease.

No.	Reference	Age (y)/sex	Diagnosis	Preceding infection	Initial neurological symptoms	Duration^a^	Immunotherapy	Clinical outcome
1	Current case	63/F	ADEM	No	Headache, ataxia	8 days	IVMP, PSL	Recovered
2	7	34/F	ADEM	No	Headache	11 days	IVMP, PSL, IVIG	Mild hemiparesis
3	8	51/F	ADEM	RTI	Headache, spatial disorientation	–	IVMP	Ambulatory
4	9	38/F	ADEM	RTI	Headache, hemiparesis	2 days	DEX, IVMP, PSL	Recovered
5	10	41/M	ADEM	RTI	Dysarthria	5 days	IVMP, IVIG	mRS score 3
6	11	18‐month‐old/F	ADEM	Febrile rash	Left hemibody seizures with fluctuating consciousness	5 days	Corticotherapy, IVIG	Discrete hemiparesis
7	12	32/F	ADEM	RTI	Hemiparesis	Few hours	Steroids	Death
8	13	37/F	ADEM	RTI	Headache	4 days	DEX	Recovered
9	14	17/M	AHL	MP infection	Difficulties in walking	–	Steroids	Paraplegia
10	15	31/M	AHL	MP infection	Right arm weakness	6 days	Steroids, PE	Recovered
11	16	7/F	AHL	No	Headache	4 days	IVMP, PSL	Demonstrate impulsivity
12	17	25/F	AHL	RTI	Headache	3 weeks	IVMP, PE	Able to walk with a cane
13	18	6/M	TDLs	No	Behavioral change, altered gait	1 month	DEX, IVMP, PSL	Recovered
14	19	50/F	TDLs	No	Partial seizures, dysdiadochokinesis	2 weeks	Steroids	Very mild dysphasia and slight paresis of the right arm
15	20	53/F	TDLs	–	–	Few hours	–	–
16	21	38/M	Marburg variant of MS	No	Right lower extremity palsy	–	DEX	Alive
17	22	31/F	Marburg variant of MS	No	Consciousness deterioration, hemiparesis	7 days	DEX, PP, IVIG	mRS score 2

^a^ Time from onset to craniectomy.

Abbreviations: ADEM, acute disseminated encephalomyelitis; AHL, acute haemorrhagic leukoencephalitis; DEX, dexamethasone; IVIG, intravenous immunoglobulin; IVMP, intravenous methylprednisolone; MP, *Mycoplasma pneumoniae*; mRS, modified Rankin Scale; MS, multiple sclerosis; PE, plasma exchange; PP, plasmapheresis; PSL, prednisolone; RTI, respiratory tract infection; TDLs, tumefactive demyelination lesions.

Although the cytokine profile in the CSF was not investigated in any of these reports, the present case was characterized by a markedly elevated IL‐6 level in the CSF. IL‐6 is a pleiotropic cytokine present during inflammation. In inflammatory diseases of the CNS, elevated levels of IL‐6 in CSF have been reported in NMO,^23^ ADEM,^24^ AHL,^25^ and progressive leukoencephalitis after SARS‐CoV‐2 infection.^26^ The clinical efficacy of satralizumab, a humanized monoclonal antibody targeting the IL‐6 receptor, has already been demonstrated in NMO in a randomized, controlled trial,^27^ and tocilizumab, another humanized monoclonal antibody targeting the IL‐6 receptor, has been reported to be effective in AHL in childhood.^28^ Although decompressive craniectomy and immunotherapy with steroids were effective in the present case, it may be important to evaluate IL‐6 levels in the CSF, and administration of IL‐6 receptor inhibitors may be one treatment option for the clinical management of fulminant demyelinating disease, considering that mortality and serious disability may accompany the disease. It is important to continue to enhance our knowledge of IL‐6 in CNS inflammatory diseases.

In summary, a case of a fulminant demyelinating disease of the CNS that required decompressive craniectomy for rapid development of cerebral herniation was presented. It is important to perform neurosurgery with immunotherapy within a reasonable period of time because fatal cerebral herniation can develop even in autoimmune diseases of the CNS within a week of onset. In addition, the IL‐6 level in the CSF was significantly elevated in this case. It is important to investigate the cytokine/chemokine profile in the CSF of cases of fulminant demyelination.

## AUTHOR CONTRIBUTIONS

**Toshihiro Ide:** Conceptualization; investigation; writing – original draft; writing – review and editing. **Ryo Ebashi:** Supervision. **Makoto Eriguchi:** Supervision. **Shinichi Aishima:** Supervision. **Tatsuya Abe:** Supervision. **Hideo Hara:** Writing – review and editing.

## FUNDING INFORMATION

This case report did not receive any specific grant from funding agencies in the public, commercial, or not‐for‐profit sectors.

## CONFLICT OF INTEREST STATEMENT

None declared.

## CONSENT

Written, informed consent was obtained from the patient to publish this report in accordance with the journal's patient consent policy.

## Data Availability

All the required information is included in the manuscript.
